# The Patients' Practises Disclosing Subjective Experiences in the Psychiatric Intake Interview

**DOI:** 10.3389/fpsyt.2021.605760

**Published:** 2021-05-10

**Authors:** Enikö Èva Savander, Jukka Hintikka, Mariel Wuolio, Anssi Peräkylä

**Affiliations:** ^1^Department of Psychiatry, Päijät-Häme Central Hospital, Lahti, Finland; ^2^Faculty of Medicine and Health Technology, Tampere University, Tampere, Finland; ^3^Faculty of Social Sciences, University of Helsinki, Helsinki, Finland

**Keywords:** psychiatric assessment interview, mental disorder, subjective experience, conversation analysis, self-disclosure

## Abstract

In psychiatric diagnostic interviews, a clinician's question designed to elicit a specific symptom description is sometimes met with the patient's self-disclosure of their subjective experience. In shifting the topical focus to their subjective experiences, the patients do something more or something other than just answering the question. Using conversation analysis, we examined such sequences in diagnostic interviews in an outpatient clinic in Finland. From 10 audio-recorded diagnostic interviews, we found 45 segments where medical questions were met with patients' self-disclosures. We show four sequential trajectories that enable this shift of topic and action. There are four possible trajectories: (1) the patient first answers the medical question and the clinician acknowledges this answer, whereupon the patient shifts to a self-disclosure of their subjective experience; (2) the patient first gives the medical answer but shifts to self-disclosure without the clinician's acknowledgement of that answer; (3) the patient produces an extensive answer to the medical question and, in the course of producing this, shifts into the self-disclosure; (4) the patient does not offer a medical answer but designs the self-disclosure as if it were the answer to the medical question. We argue that in the shifts to the self-disclosure of their subjective negative experience, the patients take local control of the interaction. These shifts also embody a clash between the interactional projects of the participants. At the end of the paper, we discuss the clinical relevance of our results regarding the patient's agency and the goals of the psychiatric assessment.

## Introduction

In contemporary psychiatry, patient assessment is guided by the classification of mental disorders. In psychiatric interviews, the clinician's goal is to evaluate the patient's problems and provide an evidence-based treatment grounded on symptom-oriented diagnostic ICD-10/DSM-5 categories (1, 2). Since the 1980s—after the emergence of the DSM-III—many researchers have pointed out that the patient's subjective experience is overlooked in the contemporary descriptive classifications. It has been argued that by attempting to define objective signs and symptoms as in the other fields of medicine, psychiatry de-contextualises, simplifies, and reifies mental phenomena. While the symptom-oriented diagnostic categories may have provided for better reliability of diagnoses [however, see Vanheule (3), who clearly argues against it], the validity of psychiatric diagnoses—something that requires the understanding of the individual psychopathology—has been neglected in clinical work and research (4–11).

Clinician–patient communication is important in psychiatry because social interaction with the patient is the clinician's primary means for understanding, evaluating and eventually diagnosing the patient's mental suffering. Communicative practises in psychiatry have been taken up in studies on shared decision-making, mutual understanding, and patients' expectations (12–17). For example, researchers showed a considerable interactional tension in routine psychiatric consultation with psychotic patients as the patients repeatedly attempt to talk about their psychotic experiences, and the clinicians reject the topic (18). Other studies have demonstrated how minute practises in clinical communication are associated with better understanding and patient adherence. The frequency of patients' requests to psychiatrists to clarify what they say is associated with better treatment adherence (16). Training emphasising the importance of understanding the patients' psychotic experiences, on the other hand, is associated with psychiatrists' increased use of self-initiated clarification as “self -repair” (19) in their talk.

Clinicians' questions are important in all medical interviews (20–22). By asking questions, the clinicians gather information about the patients' history, experiences, and symptoms, yielding diagnoses and treatment recommendations. In psychiatric assessment interviews, the role of questions is particularly significant because the psychiatrist does not have the support of other data gathering instruments such as physical tests.

Ziółkowska (23) analysed the doctor's questions in the psychiatric diagnostic interviews. Through linguistic analysis, she showed that the doctors objectified the patients' experiences, focusing on measurable symptoms or behaviours. This was achieved by the use of “nominal phrases” (such as “*will to act*” or “*thoughts about death*”) that presented the patient's experiences without agency and context. The nominal phrases were derived from standard diagnostic manual language.

Consequently, in their responses, the patients represent their own actions and subjective experiences in the same objectified way, losing their agency and meaning of the context. Another study focused on questioning practises that acknowledge the patient's subjective experience. Thompson et al. (24) argued that a particular question design—“so prefaced declarative questions,” for example “*So you feel a bit anxious*”—conveys empathy, and they showed that their frequent use is positively correlated to therapeutic alliance and adherence.

In his now classical study *The Discourse of Medicine*, Elliot G. Mishler (20) investigated a routine medical interview as an interplay of two “voices.” He stated that the “voice of medicine” has a technical focus, providing the meaning of events without personal and social context. The “voice of the lifeworld” involves a reference to the contextual, personal meaning of events and experiences. Mishler pointed out that the standard sequential structure of the interview, consisting of the doctor's question, the patient's answer, and the doctor's assessment, maintains the doctor's control of the interview and its topical content. When the patient adds to their answer's surplus content arising from the “voice of the lifeworld,” the routine sequential organisation is interrupted, causing troubles such as hesitation, gaps, or self-repair in the doctor's next turn. In the participants' attempts to achieve coherent and shared meanings, the “voice of medicine” dominates and regulates the “voice of the lifeworld.”

Barry et al. (25) elaborated on Mishler's conception of “voices” and showed multiple relationships between them. When both the patient and the doctor operated within the voice of medicine (for example, in dealing with a broken leg), and when both of them used the “voice of the lifeworld” (for example when discussing the patient's psychosocial problem), the outcome of the general practise consultation (as measured by indicators of the patient-centred perspective) was better. The outcome was worse, however, when the doctor met the patient's “voice of the lifeworld” by transferring the topic toward the “voice of medicine.”

Some studies have explored contextual differences in how clinicians respond to patients' descriptions of their subjective experiences. Comparing general practitioners' and psychiatrists' responses to the patients' emotional disclosures of depression, Davidsen and Fosgerau (26) suggested that general practitioners dealt with the patients' emotions emphatically and took a contextual approach to their problems, while the psychiatrists treated their emotional descriptions as symptoms, using their own biomedical interpretations and explanations. Hak and Boer (27) investigated how professionals receive the patients' accounts in three clinical contexts. They found an “interrogative style” in a medical interview, in which the clinician regularly proceeded to the next question without rephrasing (formulating) the patient's answer. In a psychiatric interview with a psychotic patient, there was an “exploratively oriented style,” as the professional used formulations to check and clarify the patient's fragmented lifeworld talk, thereby transforming into the diagnostic assessment. In psychotherapy, in contrast, the clinicians formulated the gist of the patient's talk collaboratively with the patient.

In psychotherapy, the therapists' responsiveness to the patients' accounts of their experiences is at the heart of the clinical task (28–31). By analysing cognitive–constructivist psychotherapy, Voutilainen et al. (29) showed that the therapist combined recognition and interpretation to access the patient's experiences. Through recognition, the therapist validated the patient's emotions, thereby preparing the interpretation of their experiences. Furthermore, by analysing cognitive psychotherapy and psychoanalysis, Weiste and Peräkylä (31) demonstrated that therapists use four types of responsive formulations to gain access into the patients' experiences. They found that rephrasing and highlighting formulations were common in both therapeutic approaches.

Nonetheless, they were relocating formulations only in psychoanalysis and exaggerating formulations only in cognitive psychotherapy. According to this study, it can be said that different therapeutic approaches apply only partially different formulations in therapeutic responses.

Thereafter, Weiste et al. (32) took up what they call the “epistemic relation” between the psychotherapist and patient, showing how the psychotherapist attempts to maintain the patient's primary right to know about and define their inner experiences. All in all, interactional studies on psychotherapy document how clinicians attend to the patients' subjective experience. Such attentiveness is something that is largely lacking in psychiatric interviews, as the studies cited earlier suggested.

While earlier research has covered some of the ways in which clinicians respond to the clients' accounts of their experiences, as far as we know, no interactional research has been made on how clients manage (or fail to manage) to insert their experience-oriented tellings in the psychiatric interview. This will be the task of the paper at hand. To understand how clients bring forth their experience in the interview, the concept of “*self-disclosure”* as social action is illuminating.

The idea of self-disclosure was introduced in the work of Canadian psychologist Sidney M. Jourard [(33), p. 19], who presented that “self-disclosure is the act of making yourself manifest, showing yourself so others can perceive you.” He also stated that self-disclosure is a sign of growing toward a healthy personality and healthy relationships and viewed its function in the treatment process of psychotherapy and psychiatry. Antaki et al. (34), reviewing it from the interactional perspective, argued that “self-disclosure is a social performance which must be brought off in interaction, and has its interactional context and its interactional consequences” [(34), p. 181]. In data of naturally occurring therapeutic conversations, they pointed out three specific features of this interactional performance. First, it is manifested voluntarily when the speaker discloses some topic initiatively by themselves. Second, it is significant because it indicates that the telling is newsworthy, highly emphasised, or coloured, for example by means of “extreme case formulations” (35). Third, the self-disclosure reports personal information of intimate experiences being possibly a “bonus,” thus over and above the momentary expectations of the co-interactant. Recently, Logren et al. (36) investigated self-disclosures in group counselling.

Recently, attempting to develop the participants' collaboration and advance patient-centred or individual evaluation in the psychiatric assessment interview, Savander et al. (37) compared two kinds of psychiatric assessment interviews: one supported by psychological case formulation (38, 39) and one following the standard medical approach (40). Clinicians who had received training in psychological case formulation asked questions about the patient's subjective experiences more frequently and topicalized such accounts more actively in the subsequent talk compared to clinicians whose interview style was based on the standard DSM/ICD orientation. Based on a quantitative analysis, this study also suggested that the patients frequently “go against the grain” in telling the clinicians about their subjective experiences. The patients offer their subjective experience accounts despite the fact that the clinicians do not invite or topicalize them.

In the work at hand, we will qualitatively examine one key environment where the patients go against the grain in reporting their subjective experiences. We focus on the patients' answers to clinicians' questions. In the cases that we examine, the clinicians' questions concern medical matters, while the patients include self-disclosures of their subjective experience in their answers.

### Objectives

We investigate the patients' possibilities and practises to disclose their negative subjective experiences in response to the clinicians' medical or factual questions. We aim to explicate the interactional practises that the patients use in doing self-disclosures of their negative subjective experiences in this conversational environment.

## Materials and Methods

### Participants and Data

Our data were initially collected for a randomised clinical study in a community mental health centre in Finland (37, 41). The study was accepted by the Ethics Committee of Tampere University Hospital. For that study, we audio-recorded 45 psychiatric intake interviews with patients who were referred to the mental health centre by their primary care or occupational health doctors. For the study at hand, we used 10 intake interviews (altogether 563 min). Five interviews represent the usual standard psychiatric interview (ICD/DSM) practise (Assessment as Usual, AAU group) (40), while the other five involve psychological case formulation based on dialogical sequence analysis (DSA group) (38, 39). While the interviews were not guided by a ready-made question list, the clinicians, especially in the AAU group, asked questions that routinely belong to the psychiatric diagnostic evaluation. The sequences that we examined came from two (often overlapping) phases of the interview: patient history and exploration of their present condition. During the interviews, some of the clinicians made notes on paper. They worked with a computer only at the end of the encounter, for example, when writing prescriptions or other formal statements. As the study at hand focuses on patients' interactional practises (rather than the clinicians' interview style), we did not separate between these two types of interviews but used all the data as one pool. We excluded patients with neuropsychiatric disorders and psychotic disorders and also those who needed acute assessment within 7 days. In each interview, the participants were two clinicians (a physician with a nurse or a psychologist) and the patient. The data from 10 interviews involved three psychiatric residents, three psychiatrists, three psychologists, three nurses, and ten adult patients (four female and six male adults) with various symptoms and diagnoses.

We audio-recorded 40 first visits in the DSA group and five randomly selected first visits from the AAU group. The five AAU interviews lasted 280 min, and the five DSA interviews lasted 283 min. Using the matching method, we chose five DSA interviews that corresponded with the five randomly selected AAU interviews. The matching was based on patient characteristics including (1) gender, (2) age, (3) educational level, (4) psychiatric treatment history, (5) substance abuse history, (6) ability to self-reflect, and (7) ability to verbalise experiences [for more details, see (37)].

For an earlier quantitative study (37), from the recorded interviews (*N* = 10), we collected all patients' utterances in which they described their negative subjective experience in non-medical terms (*N* = 119). Sometimes, these utterances were preceded by the clinician's questions which focused on experiential (nonmedical) matters and sometimes by questions that focused on medical matters. In this study, we qualitatively examine the sequences where the patient's subjective experience-oriented utterance was preceded by the clinician's medically oriented question. There were 45 such sequences.

### Procedure

The data were analysed using conversation analysis (CA). CA is a qualitative method for examining action sequences in social interaction (42). As mentioned above, the actual question–answer sequences that we analysed came from our previous study (37), where they were examined quantitatively. The sequences involved the clinician's medically oriented questions followed by the patient's response focusing on their negative subjective experiences.

The point of departure in our previous and current work has to do with the topical focus of utterances: we differentiated between *medical* and *experiential* domains. This distinction corresponds grossly to Mishler's (20) binarity between the “voice of medicine” and “voice of the lifeworld.”

The complexities pertaining to the binarity between medical and experiential domains should be acknowledged. In one sense, everything the patient tells about their symptoms and psychological problems involves subjective experience; otherwise, the patient could either not tell about psychological problems involving subjective experience or would not be able to talk about it. Likewise, all clinicians' questions have to be linked to some aspect of the patient's experience in order for the patient to be able to answer at all. On the other hand, anything that the patient tells about himself/herself can ultimately be understood as medically relevant. Our distinction between medical and experiential domains, however, is more specific. We assume that the clinician's questions are often informed by the standard agenda of psychiatric interview, their aim being to collect information for the diagnosis. Even if such agenda questions have to do with subjectively experienced things such as appetite, sleeping problems, or feeling high, we consider them as questions that belong to the medical domain. On the other hand, the patients can provide the information that was asked for and thereby remain in the medical domain—or alternatively, they can tell something else about their lives, thereby shifting to an experiential realm. Our focus was particularly on the patients' tellings about their negative subjective experiences. In the earlier study where the coding was initially done, we defined the experiential answers as ones that “the patient describes in negative terms a personal feeling, attitude, experience, or life event” [(37), p. 1297].

Thus, in our corpus, medically oriented questions involved interrogatives or inferential statements about symptoms or behaviours characterising the patient's condition or functioning. Such questions did not seek to elucidate the meanings, which the patient attributes to symptoms or behaviours. The patients' responses focusing on subjective experiences involved negatively valenced voluntary descriptions—doing more than just answering the question—of personal experiences, feelings, attitudes, or life events. In terms of action, these answers were understood as self-disclosures. In the qualitative data analysis presented in this paper, self-disclosure as action proved to be the key concept.

The preliminary analysis of the interviews was done by the first author. She identified 45 question–answer sequences that were analysed for this paper. The sequences started with the clinician's question with a medical focus, which was followed by the patient's response which included an account of negative subjective experience. Examining these sequences, the first and fourth authors found four trajectories in and through which the shift from medical to experiential focus could take place. Of the 45 question–answer sequences, 40 could be grouped in one of the four trajectories. The remaining five did not fit in any of the trajectories, and they are not analysed in this paper. In these cases, for example, either the nurse's question was not heard by the patient or the patient's experience was positively and not negatively valued. After the grouping of the sequences, the first author analysed all instances in each group. Based on this analysis, the first and fourth authors selected the clearest and most representative sequences from each group. These were subjected to in-depth conversation analysis performed by the first author and followed by elaboration by the first and fourth authors.

Most of these sequences are presented in this paper.

## Results

In all examples, the indirectly identifiable data have been presented without gender and age, and all personal information has been anonymized in the extracts. In our collection, we found four different trajectories in which the focus of the participants' talk shifts from the clinician's medical question to the patient's disclosure of their subjective experience in the following turn. Out of the 45 cases, 40 represented one or the other of these four trajectories. Before we present the trajectories, we will show an example of where the patient does *not* shift the focus of the talk after a medical question, but instead produces an answer that remains in the medical domain. This is the “baseline” trajectory, from which the cases differ from those to be shown later. Consider Extract 1.

**Figure:**
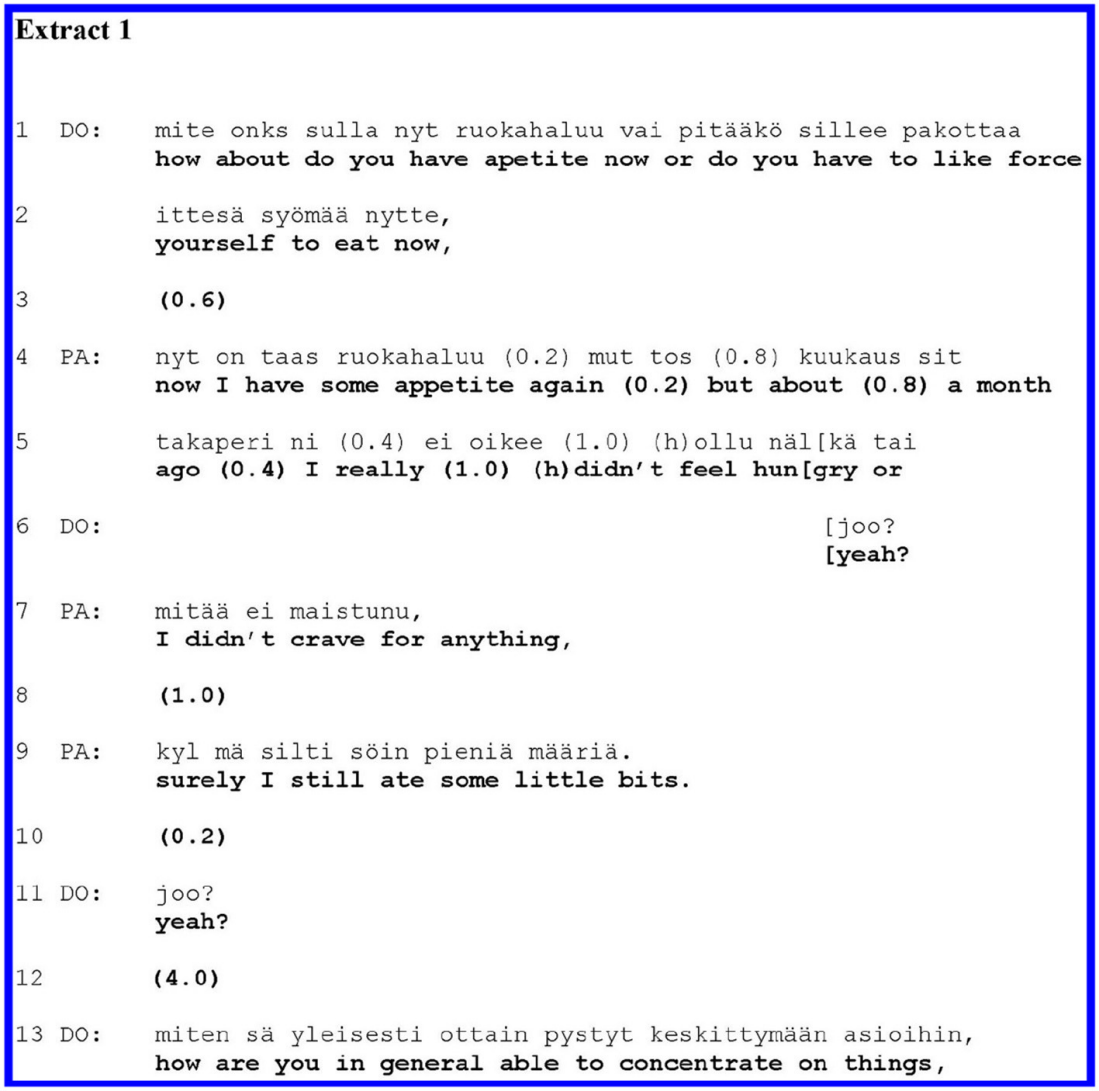


Extract 1 is a typical instance of a medically oriented psychiatric interview. In lines 1–2, the doctor (DO) asks a yes–no question about appetite. This is a paradigmatic agenda question, as lack of appetite can be a symptom of depression. The patient (PA) describes the symptoms with some pauses in lines 4–10. During PA's turn, DO offers a minimal response token, “*yeah?”* (line 6), thus encouraging PA to continue and extend the response. In lines 11–12, DO acknowledgement and a long pause (4.0) close this sequence and the topic of appetite. Maintaining medical orientation, DO asks another symptom-oriented question in line 13. PA's answer (lines 4–9) involves more than a mere “yes” or “no,” which would grammatically be the minimal adequate answer to the question. Yet in her extensions of the answer, PA remains on the topic (appetite) and indeed gives further information related to it, which hearably serves the question's agenda (information gathering about appetite). The patient does not make self-disclosures of their subjective experience. Contrary to our subsequent cases, here it is mainly the clinician who controls the interview by asking medically oriented questions.

While the question–answer structure generally allocates control of the topic and action to the questioner (20–22), in the trajectories we analysed, the patient takes some control by steering the talk. In some trajectories, the patient's “grasp” of control is more pervasive than in others. Below, we will present the four trajectories in an order related to the patient's control, starting from the weakest control and moving toward the stronger control.

### Self-Disclosure of Personal Experience After Medical Answer and Its Acknowledgement

In the first type of trajectory, the clinician's medical question is followed by the patient's answer, which focuses on the medical realm. Thereafter, the doctor acknowledges the patient's answer, and after this acknowledgement, the patient moves on to self-disclosing personal experience. The patient takes topical and action-related control as they make the self-disclosure. The grasp of control is not drastic, however, as the patient first cooperates fully with the clinician's question, offering a topically adequate answer and waiting for the clinician to acknowledge it.

We discovered 15 sequences following this pattern, of which we will introduce two cases. Extract 2 below is from an interview with a young adult patient whose main symptoms are anxiety and unspecified stomach pain. In the past, PA has suffered from a malignant disease, but the control has been normal for several years. In lines 1–4, DO explicates their plan for the rest of the interview: DO still wants to cover *some things* before moving on to discussion of treatment (lines 3–4*: “what you might benefit from”*).

**Figure:**
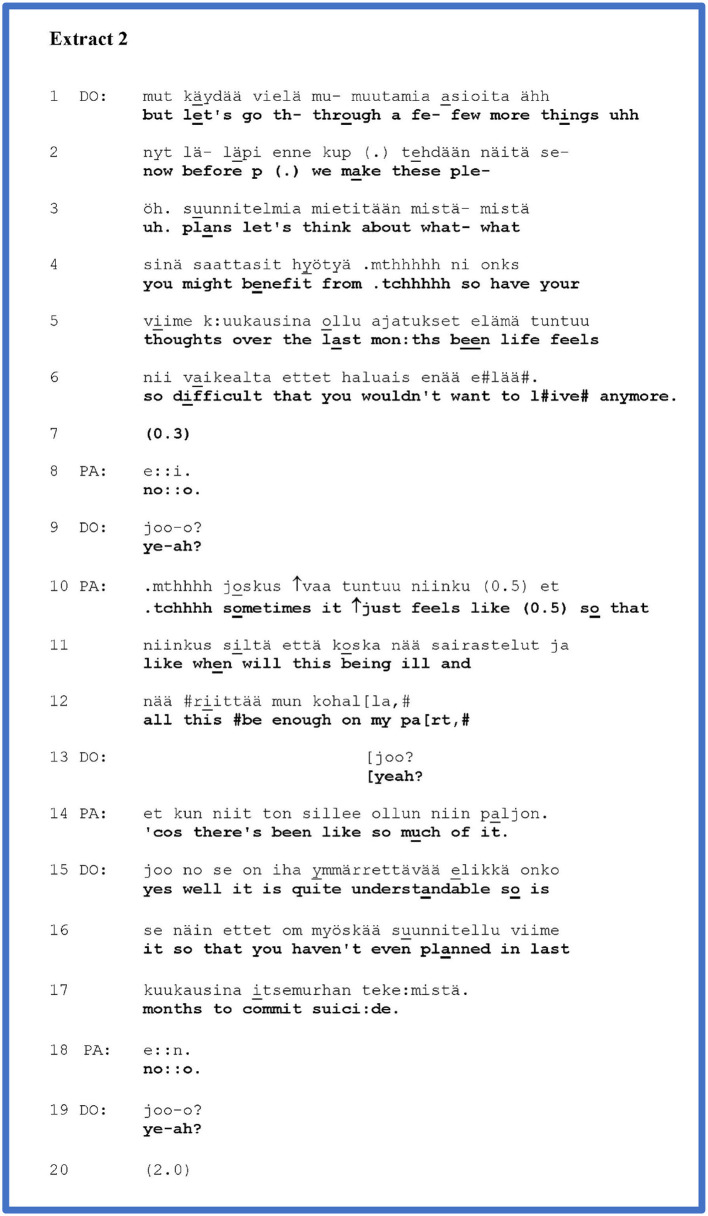


In lines 4–6, DO asks about suicidal thoughts. PA gives an answer in the negative (line 8), which DO acknowledges in line 8. After the acknowledgement, PA moves on to self-disclose a negative personal experience (lines 10–14).

A yes–no question about suicide ideation, like the one that DO asks in lines 4–6, is part of the standard and even required agenda in a psychiatric assessment interview (40). DO's orientation to the agenda is indeed manifested in their preface to the question: DO needs to go through a few things before moving on to discuss treatments (lines 1–3). DO's voice quality during the question also appears to convey an orientation to a standard agenda: a matter-of-fact and neutral tone of voice conveys an impression of reading from a questionnaire. Yet, the question is simultaneously one that potentially touches upon a most personal experience in the patient. A “no” answer could project the closing of the topic, while “yes” would project further questions about suicidal thoughts. This dual characteristic of the question (part of the medical agenda, yet also a touching experience) may prompt how PA answers it. PA gives a simple (yet a bit delayed) answer in the negative in line 8. DO acknowledges this answer in line 9. The interactional work that the token in line does is ambiguous: it could close the sequence, but with its rising intonation, it could also be heard as a “continuer” (43) displaying an expectation of further talk to come. Immediately after the acknowledgement, however, PA moves on to talk about their tiredness with physical illnesses (lines 10–14). Thus, PA expands their initial answer with a self-disclosure of their subjective experience. By their self-disclosure, PA steps away from the possible projection of the question (closing topic after “no,” further talk after “yes”) and momentarily takes some control of the topic and action. However, PA's self-disclosure (lines 10–12, 14) is linked to DO's question and PA's initial answer: it implicitly conveys a kind of hopelessness that the question was about, even though PA explicitly denied having suicidal thoughts. In their question, DO has left the door half-open in this direction, as it were. The linkage to the question is preserved by PA's word choice: in line 10, PA recycles the word “*tuntuu”/“feels”* that was in the doctor's question (line 5). DO receives PA's answer with a continuer in line 13, whereafter PA reiterates their account once more in line 14. DO receives PA's self-disclosure with a normalising evaluation (line 15), whereby they decline the possibility of further talk about it. The evaluation is seamlessly followed by a question where DO returns to the question of suicide ideation (lines 15–17). PA once again answers in the negative (line 18), and DO receives the answer in line 19 with a token similar to the one by which she received PA's initial answer in line 9. Now the token is treated by both participants unequivocally as a “sequence closing third” (42) and DO moves on to new topic and activity (line 21).

PA's self-disclosure is designed to convey a strong negative affect. This form of a rhetorical question (44) conveys the action of complaint. The pitch contour at the beginning of the account, especially the rising pitch at the word “*just”* (line 10) is typical for complaints. Emphasising the words “*sometimes”* (line 10), “*when”* (line 11), and “*enough”* (line 12) also maintains the complaining tone (45). While the creaky voice at the end of line 12 can be associated with a turn transition (46), it also seems to convey sadness in this context. In reiterating their complaint in line 14, PA emphasises the amount or frequency of their illnesses by an “extreme case formulation” (35), “*so much*.” By all these means of affective expression, the patient's self-disclosure is designed to convey the importance and emotional weight of what is being said. PA portrays the reports as a matter of concern and personal importance, thereby legitimising the move to self-disclosure.

For another example of the patient's self-disclosure following a medical answer and the clinician's acknowledgement, consider Extract 3. PA has a mood disorder. In the history-taking phase of the psychiatric interview, DO has just explored PA's manic symptoms in their adolescence and recent past. In lines 1–3, DO offers PA a medical (and uncertain) view about the severity of their recent symptoms. Following this, in lines 3–5, DO asks whether PA has had other manic episodes in their life—using the conventional Finnish euphemism “racy” for manic. Mapping past occurrences of manic episodes is part of history-taking in mood disorders. Grammatically, the question projects a “yes” or “no” answer. Yet, in the context of diagnostic interview, the possible trajectories after different answers would be different: a “yes” would call for further elaboration of the other racy episodes, while a “no” could warrant a move to the next agenda item.

**Figure:**
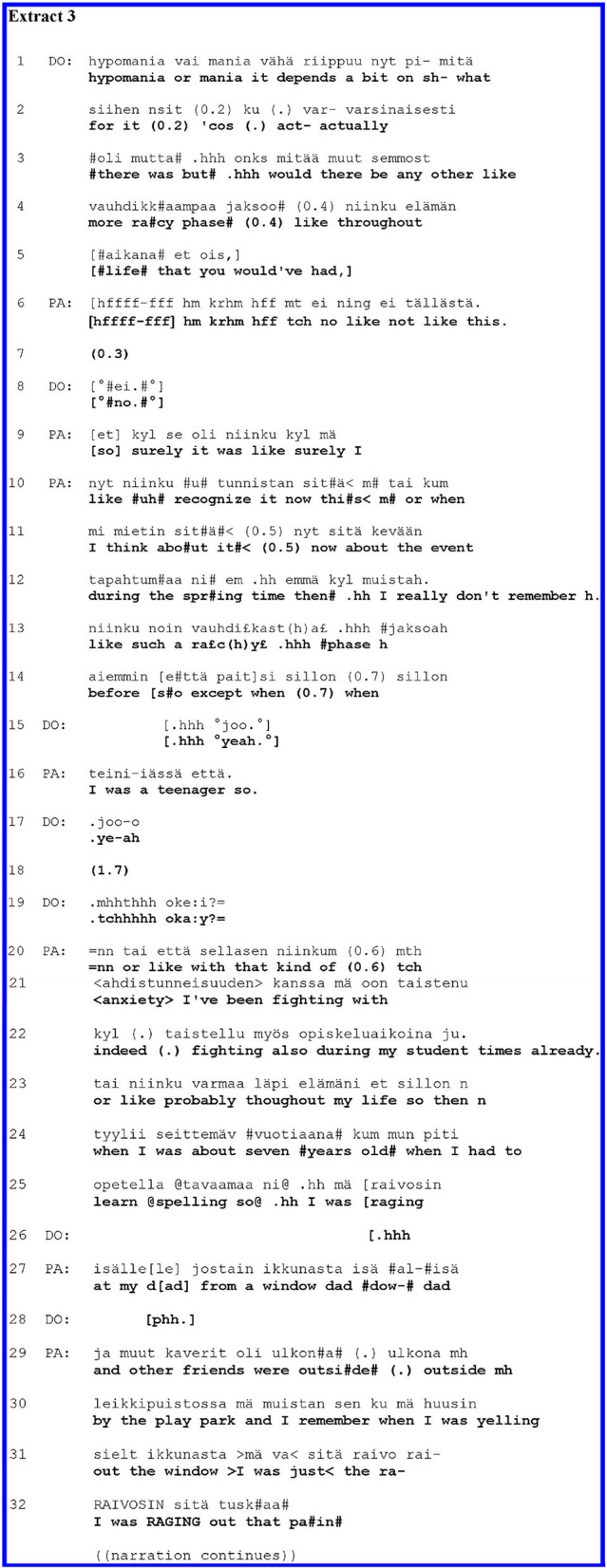


Here (as in Extract 1) PA's initial answer (line 6) orients to the yes–no polarity; in this case, the answer is in the negative, with some qualification. DO acknowledges the answer by repeating the negation word “*no”* in line 8, thereby opening up the possibility for an extension of the answer and giving the space for PA to continue. PA indeed elaborates the answer in lines 9–16, displaying something of her grounds to think that there have not been other “racy” episodes. Through the expansion, the patient orients herself to the medical diagnostic agenda: she provides information about possible specific symptoms in her past. DO receives the patient's elaboration by a string of acknowledgements (lines 15, 17, 19); the final one (“*okay”* in line 19), through its placement (after a gap following the acknowledgement in line 17) and design (rising intonation), not only closes the prior sequence but also projects a move to the next question or action (47). At this point, PA hurriedly cuts in (line 20) and moves on to extend their account with a self-disclosure of negative personal experience.

PA's answer in lines 6–16 conveyed that in their past, there have not been manic episodes other than those already discussed; however, PA now offers another view of the past, characterising it as one filled with anxiety. The change of direction in PA's account is embodied in the turn beginning “*tai että*,” best translated “*or like”* (line 20): PA seems to point out that, despite the fact that there were no more manic episodes, there still were mental problems. PA's self-disclosure is expressive, emphasising the key descriptor of the subjective experience, “<*anxiety*>,” by stressing two syllables and slow delivery. PA highlights the effort to endure anxiety by using the word “*fighting”* (lines 21–22). PA first points out that this fighting against anxiety took place during their student years, but thereafter, they upgrade the temporal characterisation with an extreme case formulation, “*probably throughout my life”* (line 23). PA continues their expressive emphasised account with a storey about a scene from their childhood; here, the repeated key descriptor, “*I was raging”* (lines 25, 31, and 32), depicts a particularly intensive negative experience. The louder volume at the final delivery of this descriptor (line 32) seems to embody PA's forceful emotions.

In sum, after the clinician acknowledged the patient's answer to the clinician's factual and medical question, the patient in Extract 3 self-disclosed a negative subjective experience on their own initiative. The self-disclosure was designed as a self-corrective expansion of the patient's medical answer.

With expressive delivery, verbs depicting intense emotions, extreme case formulation, and storytelling, the patient's self-disclosure conveys the importance and emotional weight of being said. Here, as in Extract 2, such practises seem to legitimise the patient's shift to self-disclosure.

In Extracts 2, 3, the patients' self-disclosures occurred after the clinicians' medical questions, the patients' answers, and the clinicians' acknowledgements of the answers. In these environments, the patients' shift to self-disclosure of their subjective experience is relatively fluent and direct. Nonetheless, the patients intensify the meanings of their telling, thereby seemingly legitimising their self-initiatory accounts of subjective experience.

### Shifts Without Prior Acknowledgement of the Answer

In the cases shown above, the patients shifted to self-disclosure of personal experiences after the clinicians had acknowledged their medical answers. Sometimes, however, the patient gives the medical or factual answer but then moves onto their self-disclosure *without* the clinician's acknowledgement of that answer. Thereby, the patients take somewhat more control of the course of the interaction than they do in the cases shown thus far. In our data, there were 11 sequences in this group; below, we will show one of them.

In Extract 4, PA has a mood disorder and aggression management problems. Two clinicians are present: DO and a psychologist (PS). Before PS's question, PA complained about some physical symptoms associated with his anger (not shown here). Thereafter, in line 1 of the extract, PS asks a follow-up question on the topic of anger.

**Figure:**
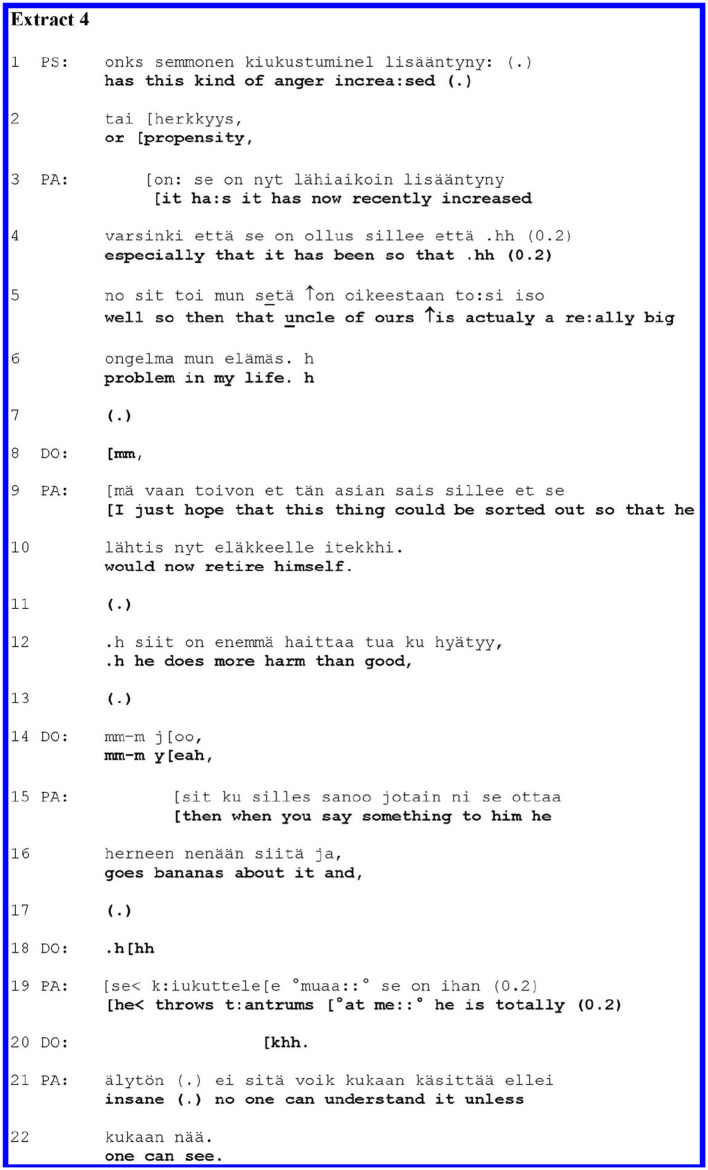


PS's question (lines 1–2) seeks to clarify whether PA's anger or propensity for it has increased. Here the anger is a medical symptom expressed as a noun without context, and the clinician asked about its magnitude. The polar question projects a “yes” or “no” answer; a “yes” could particularly make relevant further inquiries or elaboration about the anger. The patient gives an answer in the positive and moves on to extend their turn with a self-disclosure of problematic personal experience (lines 3–22).

PA's turn initiation (line 3) overlaps with the end of PS's question. The overlap may imply that the answer is designed as one that arises from PA's own perspective (48–50). PA first answers with minimal confirmation, “*it ha:s*,” whereafter PA redoes and specifies the confirmation in the same prosodic unit by a sentence where PA recycles the verb from the question, “*it has now recently increased especially that it has been so that*.” This two-fold turn-design (minimal confirmation plus elaboration that recycles the key term from the question) may adumbrate an independently articulated and expanded account where PA would break away from the terms of the question (51–53). However, the specification of the confirmation is left incomplete, as PA aborts their sentence construction, breathes in, pauses (line 4), and restarts with a new sentence in line 5. Here, PA self-discloses a personal experience, bringing in a new but related topic—problems with their uncle. Naming a problem (lines 5–6) projects its further unpacking and elaboration (54). PA indicates the intensity of the problem by extreme case formulation: “*actually a re:ally big problem in my life h*.” DO aligns themselves as a recipient of such elaboration by the minimal response particle “*mm”* in line 8.

PA extends the self-disclosure by elaborating the complaints regarding their uncle in lines 9–22. The emotional intensity of their account is encapsulated, for example in the idiomatic depiction of the uncle's unreasonable reactions, ‘*ottaa herneen nenään*,” which could possibly be translated as “*goes bananas*,” and in characterisation with an extreme case formulation: “*he is totally (0.2) insane*.” DO remains in the recipient's position, as indicated by the response particles in line 14.

In Extract 4, the patient first answers minimally to the clinician's medically oriented question. Thereafter, in two “steps,” the patient shifts to a self-disclosure of their problematic personal experience. The first step involves redoing (lines 3–4) the initial answer so that it adumbrates the self-directed talk. The second step involves self-interruption and restarting, leading to the self-disclosure where they complain about the uncle. In describing their problems with the uncle, the patient employed expressive and emphasising language, which seemingly legitimised the move into self-disclosure.

By shifting to their self-disclosure of personal experience without waiting for the clinician's confirmation of their initial answer, the patient took more of the local control of interaction than the patients in Extracts 2, 3. Common to all the above extracts, however, were the expressions of intensity of concern that legitimised the shift to a self-disclosure of personal experience.

### Shifts to Self-Disclosure Within the Patients' Extended Response

In the extracts shown thus far, the clinicians' medical questions were followed first by a medical answer, whereafter the patient shifted to a self-disclosure of personal experience. The clinician's acknowledgement preceded such a shift in the second and third extracts, whereas in the fourth one, the patient made the move without the intervening acknowledgement. We discovered nine sequences following more complex patterns, of which we will introduce one case. In Extract 5, shown below, the patient produced an extended answer to the medical question, and here, the medical and experiential realms were intertwined throughout the answer.

The patient concluded by self-disclosing a problematic experience, yet there was not a definite point where the medical answer ended and the self-disclosure began. Rather, the shift toward a self-disclosure of personal experience involves what has been called “stepwise transition” (55).

**Figure:**
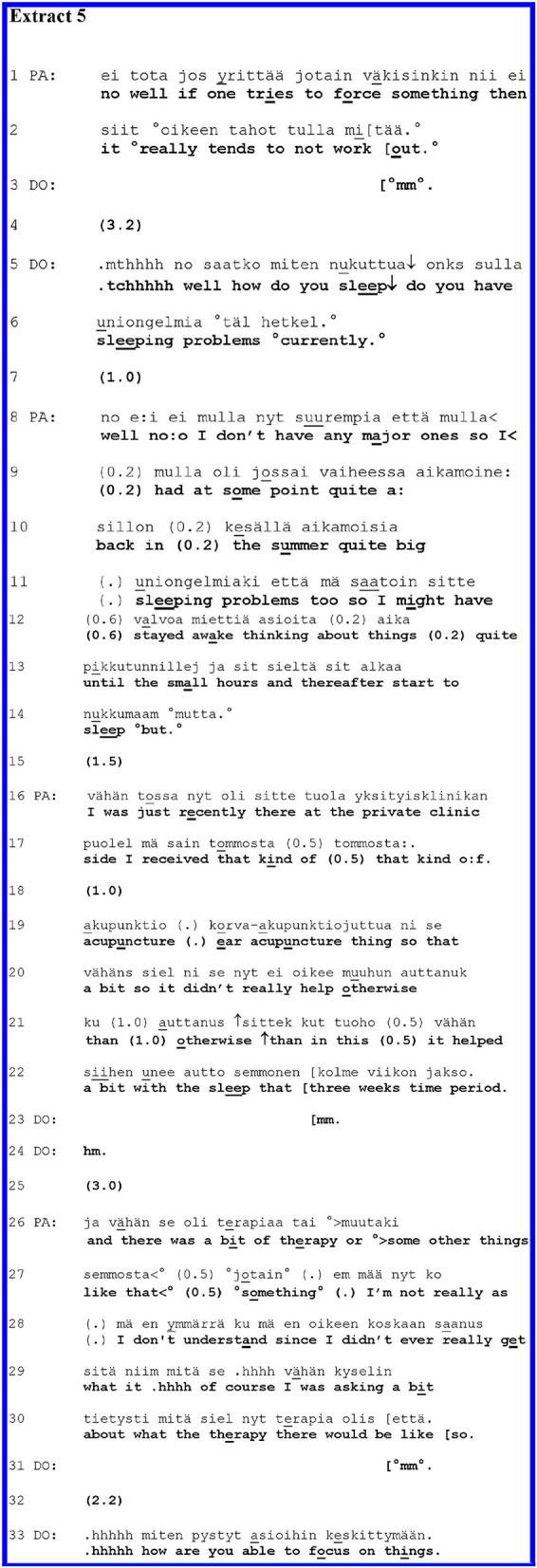


Extract 5 highlights a middle-aged PA with a recurrent mood disorder and features of personality pathology. The extract is from the history-taking phase of the interview. Prior to the extract, PA had been telling about their tiredness and nervousness. The end of this telling is shown in lines 1–2. DO receives this with a softly uttered “*mm”* (line 3). After a long gap in line 4, DO asks the next question about sleeping difficulties.

DO's question in lines 5–6 seems to have a double function in this context: on the one hand, it shifts to a new standard agenda item (sleeping); on the other hand, it is tied to the previous discussion about tiredness and nervousness, which might lead to sleeping difficulties. This double function is reflected by the structure of the turn, which consists of two parts. The first part involves idiomatic sentence structure combining “yes–no” and “Wh” question features (translated here as “*how do you sleep?”*). With this question, DO invites an evaluation of PA's sleep; this evaluation might be tied to the prior talk about tiredness and nervousness. DO, however, continues with another interrogative sentence, which is more specific and oriented to standard medical agenda: a polar question about “*sleeping problems”* in the present time.

In the extended answer beginning on line 8, PA moves gradually from the factual, medically oriented answer through stepwise topical transitions to self-disclosures of personal experience; they end up with complaints about not having received psychotherapy.

After a 1.0-s gap, PA starts the response in line 8, targeting the latter part of DO's question by hesitantly stating, “*well no:o*,” whereafter PA continues with the qualification, “*I don't have any major ones*.” This is followed by beginning a further elaboration, “*so I”* (line 8), which then, after a pause, leads into a narrative in lines 9–14 about having prior sleeping difficulties. In and through the answer, PA moves from the initial answer in the negative, “*well no:o:*,” toward an assertion of problems having been there, and from focus on the present to focus on the past. Furthermore, PA moves from the categorical answer (“*no”*) to a self-initiatory characterisation of the sleeping difficulties that PA has had (in lines 9–14). PA emphasises the severity of their sleeping problems by characterising them as “*quite big”* (lines 9–10) and pointing out that PA stayed awake “*until the small hours”* (line 13). In this self-initiatory elaboration of their answer, the patient still orients themselves to the agenda of the question, providing information about the sleeping problems. Yet, by describing the details of the problems (thinking about things until the small hours), the patient also makes a move toward the self-disclosure of personal experience.

In line 14, PA's turn seemingly trails off. PA ends the utterance with the connector “*but”* which might also project further talk, possibly disjunctive talk. DO seems to be alive for the possibility of further talk and remains silent, and after a gap, PA resumes their account in line 16. Now PA tells about ear acupuncture therapy in a private clinic, which helped with the sleeplessness “*a bit”* (lines 19–20).

While acknowledging this help, however, PA points out that the acupuncture did not help “*otherwise”* (line 21). DO acknowledges PA's telling by minimal “*mm*” tokens in lines 23 and 24. After a silence of 3.0 s (line 25), PA produces yet another extension of the account, moving hesitantly on to something that is hearable as a complaint for not having received “*therapy”* at this other private clinic (lines 26–30). (Elsewhere in the interview, it becomes clear that PA complains persistently about not having been offered psychotherapy.) In lines 29–30, PA talks about asking for therapy. PA's report trails off at the end of line 30 with the final conjunction, “*että. / so*.,” which may leave some tacit meaning of the previous topic in the air, while still completing the turn (56). In line 31, DO acknowledges PA's account by “*mm*,” and after a gap, DO continues the medical agenda about another medical symptom without taking up or topicalizing PA's extended account.

In Extract 5, the clinician's complex question opened up a space for both factual, agenda-oriented information about sleeping problems, and for more contextual talk about them. The patient starts their response with a factual, medically oriented answer (line 8). Then, the patient gradually transfers the topic from the sleeping difficulties toward the last theme about the therapy in three moves, which together constituted a stepwise topical transition (55). In terms of action, PA gradually shifts from answering to self-disclosing a personal experience. During the patient's telling, the clinician positioned themselves as a passive recipient by producing quiet “*mm”* tokens (57) and remaining silent at transition relevance places. The clinician's passivity facilitates the patient's moves toward self-disclosure.

In the first transition (lines 8–10), the patient moved from claiming that they have no “major” sleep problems to accounting for their past difficulties with sleep.

In the second transition (lines 17–19), the patient moved on to talk about the ear acupuncture treatment, characterising it as having helped only “*a bit”* with the sleeping problems. Finally, in the third topical transition (lines 25–30), the patient leaves behind issues directly linked to sleep and delivers a complaining self-disclosure about not having received “*therapy*,” which is coached by the report of having asked for it at the other private clinic. Thus, the patient has moved from the medical question step by step into the self-disclosure of their negative personal experience.

In Extract 5, shown above, and in the others of this group of trajectories, the patients produced extended answers to the medical questions, and in these answers, the medical and experiential topics were usually intertwined. Likewise, two actions—answering and self-disclosure—were overlapping. In most of these cases (as in Extract 5), the clinicians' questions had two facets, as it were: while they made relevant medical and factual answers, they also left the door open to descriptions of the patients' experiences. The patients then could respond to both facets of the questions. In most cases, the clinicians remained passive and receptive, which facilitated the patients' moves toward self-disclosures of problematic experiences. In their self-disclosures, the patients highlighted by turn design the emotional intensity of the matters that they spoke about. They also took control of the conversational space more than in the extracts shown earlier.

### Shifts With the Patient's Evasive Response

In all cases shown thus far, the patients first responded to the clinicians' medical question with a medical answer, and then, in one way or another, they moved onto a self-disclosure of a personal experience. There were, however, a relatively small number of cases—only five—in which the patient did not offer a medical answer at the beginning of their turn but instead started straightaway with a self-disclosure. Only later on during their utterances, the patients may have provided a factual or medical answer. Extract 6 below is one case from this group. Through an initial question reflecting the psychiatric interview agenda, the clinician seeks information about the patient's free-time activities. The patient responded with a complaint storey about their uncle, linking that to the question but without producing a recognisable proper answer.

**Figure:**
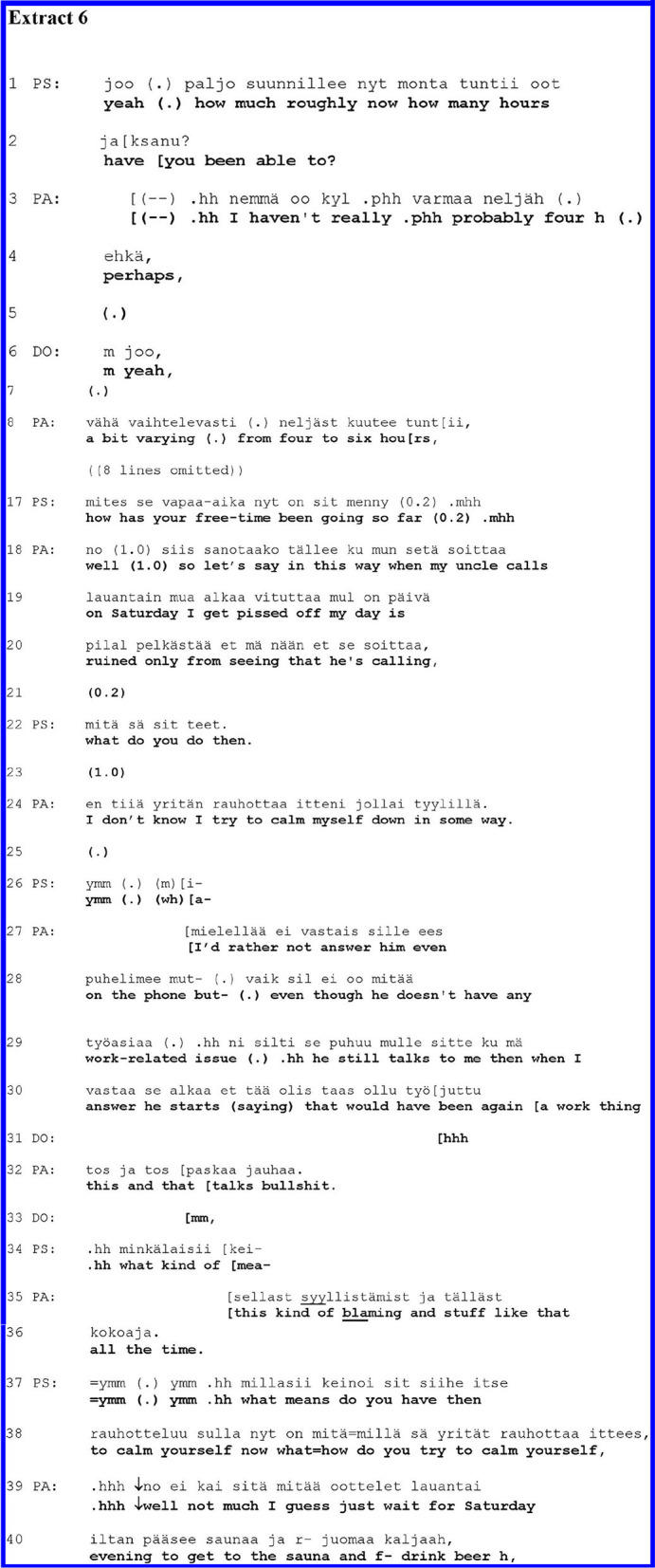


PA is the same adult as in Extract 4. This sequence is taken from a later moment in the interview. Before the extract below, the participants were talking about the patient's work-related stress. In lines 1–8, PS and PA talk about how many hours per day PA is able to work. In the omitted data (lines 9–16), PA tells about the compulsive nature of the work.

After having dealt with PA's work situation, PS asks a question in line 17 about PA's free time. Mapping out the patient's global functioning, the question about free time is a standard agenda item in the evaluation of the clinical significance of mental problems (40). Here, the question is also anchored in its local context. It follows the discussion about PA's reduced working hours (due to their health): PS now invites PA to tell increased free time. Grammatically, this question is different from the ones shown in the prior extract; as a “Wh” question (and not a yes–no question), it leaves it up to PA to decide the direction of the answer. Yet the design of the question (“*How has your free time been going?”*) invites an evaluative answer, conveying that the free time has been going well, or not so well.

Rather than offering a general evaluation of their free time, however, PA responds by telling a complaining storey about their uncle, whom PA depicts as somebody that spoils Saturdays. The storey about the uncle spoiling the Saturdays conveys an evaluative perspective of free time (made relevant by the question): Saturdays spoiled by the uncle are part of the free time. Yet, action-wise, the storey involves the self-disclosure of a subjective experience. PA starts the turn with a “*no / well”* preface, which may implicate the indirectness of the response to come and a need to negotiate the topic of the question, implying the speaker's own perspective (58, 59). After the preface and a 1.0-s pause, PA frames the answer with a particular phrase, “*so let's say in this way*,” adumbrating an answer that is not a direct response to the question but is nevertheless tied to it. After this, PA moves directly onto a self-disclosure of a personal experience, a complaint storey about the uncle who disturbs and irritates the patient with phone calls. In line 19, PA depicts the irritation by using the colloquial expression, “*alkaa vituttaa / I get pissed off*,” and characterises the consequences of the phone call using the self-pitying expression, “*my day is ruined*.”

PS aligns themselves as a recipient with a follow-up question about PA's behaviour in response to the uncle's disruptive phone call (line 22). Somewhat hesitantly, PA tells about trying to calm themselves down (line 24). In line 26, PS acknowledges PA's answer and begins something that appears as a further question about the matter. PA, however, continues the answer (describing their response to the phone calls) in lines 27–28. In line 28, PA returns the focus on the uncle and his inappropriate behaviour. The emotional tenor of the account is intensified in line 24, as PA depicts the uncle's talk as “*paskaa / bullshit*.”

In line 34, PS starts a question once more but again aborts it as PA continues telling about the uncle's bad behaviour toward PA, completing it with an extreme case formulation (“*all the time*.”). Only after two acknowledgement tokens in lines 37–38, PS returns to the aborted question (line 34) and asks to specify a means of calming themselves down. PA names the sauna and beer as the means for that.

In Extract 6, shown above, the patient answers the clinician's question with a self-disclosure in the form of a complaint storey. With a particular preface (line 18), the patient framed the self-disclosure as an answer to the clinician's question. The clinician was an active recipient asking follow-up questions, yet the patient ignored the psychologist's efforts to ask questions twice (lines 27 and 35), instead continuing the storey. In this storey, the patient portrayed the emotional weight and urgency of what they were saying in many ways: by colloquial expressions of extreme negative feelings, extreme case formulation, complaining tone, and also by overriding some of the clinician's efforts to ask follow-up questions.

## Discussion

Using conversation analysis, we investigated 10 psychiatric assessment interviews, zooming on sequences where the clinician's medically oriented question was met by the patient's self-disclosure of a problematic personal experience. We analysed the patient's means for redirecting the talk toward the self-disclosure. We found four different trajectories leading from the medical question to the self-disclosure of a problematic experience. In the first trajectory, the patient answers the medical question first, and then the clinician gives an acknowledgement, after which the patient moves on to self-disclose a problematic experience. In this trajectory, the shift to self-disclosure was relatively fluent and collaborative. In the second trajectory, the patient first gives a medical answer, and immediately after that, without the clinician's acknowledgement, she/he moves on to the self-disclosure. In the third trajectory, the patient responds to the medical question with an extensive telling in which the medical and the experiential worlds were intertwined without a single boundary between them and between the action of answering and the action of self-disclosure. In the fourth trajectory (with the smallest number of cases), the patient's turn after the medical question started off as a self-disclosure of a problematic experience, which also served as the answer.

Earlier conversation analytical research suggests that control is an indispensable aspect of social interaction: each speaker, through their turn at talking, defines and restricts the relevancies of the next turn (60, 61). In institutional contexts, the dynamics of control may be asymmetric so that one participant has more rights to control than the other (62, 63). Yet, for there to be interaction, the control can never be fully one-sided. While much of the interaction in a psychiatric interview is controlled by the clinician, in the cases presented above, the patients themselves took some control of the topic and action. In the first trajectory, the shift toward self-disclosure was relatively fluent and collaborative as the patient moved to self-disclosure only after the clinician had closed the prior action. In the second trajectory, the patient exerted somewhat more interactional control than in the first one, but the patient's move was more unilateral. In the third and fourth trajectories, the patient gained even more control; patient control was strongest when they bypassed the relevancies of the question (fourth trajectory).

It is a “default” pattern in our data that the patient responds to medical questions with medical answers (like in Extract 1). So, the presented cases are special: in these cases, the patients indeed shifted from answering the medical question to self-disclosing their subjective experience. In several of these cases, there was certain ambiguity in the clinician's question; being designed for the collection of diagnostically relevant factual information, it nevertheless left the door partially open for patients' broader accounts of their lives and experiences. This was the case in different ways, especially in Extracts 2, 5, 6. Yet, in all cases, the patients needed to do particular interactional work for the self-disclosures.

In their interactional work for the self-disclosure, the patients emphasised and intensified their descriptions of the negative subjective experiences and justified it by highlighting the intensity of their concern. The telling was thereby framed as a matter of urgency. The means for depicting the intensity of the experience and the urgency of telling included extreme case formulations, expressive (and sometimes rude) words and idioms, a loud voice, complaining tone, dramatisation through storeys, rhetorical questions, and overriding the clinicians' efforts to take a turn.

In our data, we found Extreme Case Formulations (ECFs) in almost all cases where the patients responded to medical questions with their self-disclosure of subjective experience tellings. As described by Pomerantz (35), the ECFs took diverse grammatical forms, all of which contributed semantically to extreme meanings. For Pomerantz (35), complaining was one of the key action environments of ECF [see also (64, 65)]. The patients' actions in our data—broadly, reporting their negative subjective experiences—were indeed reminiscent of complaining. The clinicians' medical questions give relevancy to a description of a patient's life and circumstances in terms of specific symptoms or other indicators of the client's medical status (22, 23, 47). In their answers, the patients might “push back” with self-disclosures justified by extreme case formulations (34) and with other practises of emphasising the matters under discussion as their “*investment”* (66, 67).

In all our extracts, we observed something we could call a “clash of interactional projects.” Schegloff (42) points out that in interactions, there are orientations that persist over sequences.

Elaborating on Schegloff's idea, Levinson [(68), p. 127] characterised such orientations: “actions often form a part of a larger project inheriting part of their import from the larger whole.” Now, the clinicians' questions in the sequences that we investigated can be understood as part of a project of gathering information for diagnosis. These questions, and how the clinicians deal with the patients' answers, serve to delineate the patient's symptoms and behaviours that may be indicative of their (assumed) underlying mental illness. The patients' project in our sequences is observably different: it is to share, and in most cases, to complain about adverse experiences. In the very sequences that we have shown, the two divergent projects meet, and the participants negotiate them. Importantly, Levinson (68) suggests that the projects often remain incomplete, and participants may also remain relatively unaware of each other's projects. As we have shown, the patients pursue their projects of complaining, despite their divergence from the clinicians' project of diagnosing; to do so, they need to resort to the interactional practises that we have shown in this paper.

In light of the idea of interactional projects, we can further ask what the patients might be seeking and possibly achieving by giving self-disclosures of subjective problematic experiences in a psychiatric interview. If the patients' project is to share or complain about adverse experiences, the response that they seek is affiliation and empathetic understanding (29, 30, 32, 69–71). Yet, in some cases, a patient's self-disclosure can also be potentially functional for the clinician's project of gathering information for diagnosis. The self-disclosure can bring out relevant additional details of the patient's mental problems. For example, in Extract 3, the patient highlighted their lifelong anxiety rather than just naming another period of manic symptoms (which would have been relevant to the question); this suggests that the clinician's project of diagnosing a possibly bipolar disorder might not actually match with the patient's clinical condition.

In this paper, we have shown the various and variable ways in which patients take partial and momentary control of the conversation in presenting their accounts on negative subjective experiences. Now, in light of what is known about psychiatric disorders, it is possible that patients with different mental disorders have different abilities in exerting control in social interaction. In some cases, personality disorders can be associated with tendencies to control interaction (1, 2, 72). We assume that patients in (hypo)manic states would be equally prone to control the interaction. In contrast, patients with depression might be much less prone to exert interactional control, at least in terms of manifest and active control. The patient in Extract 2, who was diagnosed with a mood disorder, gave their account of a subjective experience less forcefully than how the patient in Extracts 4, 6 (who has features of personality pathology) tells about the uncle; that patient first waits for the clinician's acknowledgement of the factual answer and only thereafter proceeds with the telling. In our study, these patients found certain means for disclosing their subjective experiences; they were able to do it. However, patients who are vulnerable, anxious, shy, and helpless are possibly less able to reveal their inner subjective experiences. As recognised frequently in clinical work, patients with depression or, for example, with social anxiety may feel shame and fear about stigmatisation. They have difficulties expressing themselves and telling about inner experiences, and therefore, the clinicians should seek ways to encourage them to speak.

In future studies, the possible associations between the type of mental disorder and the ability to control interaction should be examined systematically.

One should bear in mind that in our data, there are numerous cases where the patients do *not* go against the grain by inserting their self-disclosure of subjective experience descriptions in utterances that follow medical questions. It would be a topic of further study to find out what kinds of disorders—possibly severe depression and anxiety disorders—are associated with such passivity of the patients.

In this study, we focused on the patients' way of finding conversational space for the self-disclosure of negative subjective experience accounts. We made only passing observations of another equally important topic: how the doctors in the “third position” take up these accounts. In our earlier study (37), we quantitatively compared two kinds of psychiatric assessment interviews: those supported by a psychological case formulation and the standard symptom-oriented interview. The study showed that the clinician using a case formulation-supported interview took up the patient's account of a negative subjective experience with follow-up questions about the “experiential” topic in 90% of the cases; however, the clinician using standard symptom-oriented interviews took the experience up only in 40% of the cases. Earlier qualitative studies in psychiatry suggest a similar pattern where the clinician's response to the patient's subjective or emotional account tends to be poor, neutral or medically oriented (26, 27, 73).

If the clinician takes up the patient's self-disclosure with follow-up questions, there is a possibility to *understand* (and display the understanding) the patient's experience. The definition of mental disorders in the ICD-10 is a “clinically recognisable set of symptoms or behaviour associated in most cases with *distress* and *with interference with personal functions*” (2). Self-disclosures offer the clinician an opportunity to recognise and understand the patient's symptoms and behaviour associated with distress, searching the clinically significant border between mental health and disorder. With empathetic and understanding responses after the self-disclosures, clinicians might avoid the unnecessary medicalization of the patient's mental condition. Therefore, a critical task of future studies is to qualitatively investigate the clinicians' responses to the patients' self-disclosure in detail using CA.

### Limitations

In our study, the number of patient interviews and the variety of mental disorders affect and limit the significance of our results to some extent. By applying Misher's (20) binary distinction between medical and experiential orientations of the action sequences of interviews, we simplified the process of the assessment interviews. However, in that way we have been able to advance the analyzability of the data in this institutional context. We recognise that the medical and experiential realm of the interviews are more intertwined than the binary distinction represents and than the medical or diagnostic realm generated from the larger experiential realm. Furthermore, these analysed micro-sequences do not cover all details of the whole assessment interviews. However, as recently mentioned, it will be a task to investigate the clinicians' responses to the patients' disclosure of experience-oriented or medical tellings in detail using CA in the future.

## Conclusion

The patient's narrative always involves the cultural and social background as well as the actual context. The patients are seeking acceptance and understanding for their subjective experiences. In this study, we showed that after the clinicians' symptom-oriented medical questions in the psychiatric interviews, the patients actively “go against the grain” to uncover and justify their self-disclosure of subjective experiences with their social and personal context. Patients in our data found the means to disclose their subjective problematic or distressing experiences, yet they needed to work interactionally, for example by extreme case formulations, to legitimise the topical shift toward self-disclosure of subjective experiences. By being aware of and recognising the patients' relevant needs to disclose their subjective problematic experiences, the clinicians may promote and clarify the patients' diagnostic psychiatric assessment process in a patient-centred way, advancing to find the clinical border between mental health and disorder and collaboratively improving the individual treatment plan.

## Data Availability Statement

The datasets presented in this article are not readily available because the ethics committee does not allow the transfer the audio-recorded data to third parties. Requests to access the datasets should be directed to the corresponding author. Requests to access these datasets should be directed to Enikö Éva Savander, eniko.savander@phhyky.fi.

## Ethics Statement

The studies involving human participants were reviewed and approved by Ethics Committee of Tampere University Hospital (R 14094). The patients/participants provided their written informed consent to participate in this study.

## Author Contributions

ES: study design, data collection, qualitative data analysis, outlining the argument of the paper, writing the paper's first draft, and manuscript revision. JH: study design, manuscript comments, and revision. MW: qualitative data analysis, translation of extracts, manuscript comments, and revision. AP: qualitative data analysis, outlining the argument of the paper, manuscript revision, research supervision, and writing. All authors have approved the final manuscript.

## Acknowledgements

We wish to thank Emeritus Professor Mikael Leiman, Pekka Borchers M.D., Ph.D., and Melisa Stevanovic, Ph.D., for their valuable comments on the early versions of the manuscript. Furthermore, we want to thank all the participants and the staff and management of Päijät-Häme Central Hospital Department of Psychiatry for enabling this study.

## Conflict of Interest

The authors declare that the research was conducted in the absence of any commercial or financial relationships that could be construed as a potential conflict of interest. The reviewer JR declared a shared affiliation with one of the authors JH to the handling editor at time of review.

## Appendix

**Table:**

Transcription symbols—adapted from Jefferson (74)	
AB:	Speaker identification: doctor (DO), patient (PA), psychologist (PS)
→	Line containing phenomenon discussed in text
[ ]	Overlapping talk or anonymization
=	No space between turns
(.)	A pause of <0.2 s
(1.0)	Pause: silence measured in seconds and tenths of a second
°word °	Talk lower volume than the surrounding talk
WORD	Talk louder volume than the surrounding talk
.hh	An in breath
hh	An out breath
mt, tch, krhm	Vocal noises
£word£	Spoken in a smiley voice
@word@	Spoken in an animated voice
#word#	Spoken in a creaky voice
wo(h)rd	Laugh particle inserted within a word
((word))	Transcriber's comments
( )	Transcriber could not hear what was said
Word	Accented sound or syllable
-	Abrupt cut-off of preceding sound
:	Lengthening of a sound
>word <	Talk faster than the surrounding talk
<word>	Talk slower than the surrounding talk
word<	Sharp tone at the end of a word
↑↓	Rise or fall in pitch
?	Final rise intonation
,	Final level intonation
.	Final falling intonation
